# Predictive biomarkers and potential drug combinations of epi-drugs in cancer therapy

**DOI:** 10.1186/s13148-021-01098-2

**Published:** 2021-05-17

**Authors:** Tianshu Yang, Yunkai Yang, Yan Wang

**Affiliations:** 1 Beijing Key Laboratory of Cancer Invasion and Metastasis Research, Department of Biochemistry and Molecular Biology, School of Basic Medical Sciences, Capital Medical University, Beijing, 100069 China; 2 State Key Laboratory of Molecular Oncology, National Cancer Center/National Clinical Research Center for Cancer/Cancer Hospital, Chinese Academy of Medical Sciences and Peking Union Medical College, Beijing, 100021 China

**Keywords:** Epi-drugs, Biomarkers, Combination trials, Anti-tumor effect, Natural drugs

## Abstract

Epigenetics studies heritable genomic modifications that occur with the participation of epigenetic modifying enzymes but without alterations of the nucleotide structure. Small-molecule inhibitors of these epigenetic modifying enzymes are known as epigenetic drugs (epi-drugs), which can cause programmed death of tumor cells by affecting the cell cycle, angiogenesis, proliferation, and migration. Epi-drugs include histone methylation inhibitors, histone demethylation inhibitors, histone deacetylation inhibitors, and DNA methylation inhibitors. Currently, epi-drugs undergo extensive development, research, and application. Although epi-drugs have convincing anti-tumor effects, the patient’s sensitivity to epi-drug application is also a fundamental clinical issue. The development and research of biomarkers for epi-drugs provide a promising direction for screening drug-sensitive patients. Here, we review the predictive biomarkers of 12 epi-drugs as well as the progress of combination therapy with chemotherapeutic drugs or immunotherapy. Further, we discuss the improvement in the development of natural ingredients with low toxicity and low side effects as epi-drugs.

## Introduction

Epigenetics studies heritable changes in gene expression are mainly reflected in DNA methylation, histone modification, and chromosome abnormalities and are not due to changes in nucleotide states. Faulty epigenetic reprogramming composes a fundamental part of tumor cell proliferation, escape, intratumoral heterogeneity, and acquisition of therapeutic drug resistance. Accordingly, the genomic alterations and gene transcription abnormalities induced by epigenetic aberrations can have reflective effects in multiple pathways, such as EMT, Hippo signaling, p53 pathway, AMPK signaling, and cellular senescence, which can lead to the induction and maintenance of various cancers [1–5]. Emerging epigenetic drugs (epi-drugs) target enzymes involved in the regulation of aberrant epigenetic modifications in tumors. Further, epi-drugs are usually small-molecule inhibitors that inhibit key enzyme activity [6, 7]. The enzymes involved in aberrant epigenetic processes include DNA methylation-modifying enzymes and histone-modifying enzymes, such as DNA methyltransferases (DNMTs), histone methyltransferases (HMTs), histone demethylases (HDMs), and histone deacetylases (HDACs). At present, epi-drugs that are DNMT inhibitors and HDAC inhibitors are widely developed in preclinical research and clinical applications. Precision medicine paradigms provide hope for the research and clinical application of new representative epi-drugs, including the continuous development of HMT inhibitors, protein arginine methyltransferases (PRMT) inhibitors, and enhancer of zeste homolog 2 (EZH2) inhibitors [8, 9]. Furthermore, targeted drug administration based on different patient biomarkers provides a basis for precision medicine of epi-drugs. One-size-fits-all drug administration should not be adopted in clinical trials; rather, application according to the intratumoral heterogeneity should be preferred. For example, the HDAC inhibitor abexinostat can be administrated selectively according to the *Xist* expression of breast cancer patients. The use of *Xist* expression as a biomarker in this instance can improve drug sensitivity [10].

Epi-drugs are more widely used in hematological tumors than in solid tumors. Moreover, the efficacy of epi-drugs in solid tumors is still limited, which may be due to the high degree of cellular differentiation and intratumoral heterogeneity of solid tumors [11]. The intratumoral heterogeneity is observed in colorectal cancer; there are significant heterogeneity in pattern of DNA methylation in colorectal tumors, which associates with times of relapse-free and overall survival [12]. Tumor heterogeneity broadly means that tumors contain cell subsets with different phenotypes. With the development of single-cell sequencing technology, we have gradually realized that through the screening of different cellular surface markers, biochemical metabolism, and other tumor cell aspects, tumor cells are divided into subsets, and in-depth insight into tumor heterogeneity is achieved [13, 14]. By understanding the different levels of tumor heterogeneity, precision therapy has attracted more and more attention to the medical treatment in clinical oncology. Due to the genetic instability and intratumoral heterogeneity of malignant tumors, it is of great clinical significance to understand the biomarkers targeted by epi-drugs in various types of tumors.

Since reversible epigenetic abnormalities can partially lead to the continuous evolution of cancer cells, it is necessary to consider not only the precise treatment with epi-drugs but also the combination of epi-drugs with chemotherapeutic drugs or immunotherapy. The combination of epi-drugs and other types of drugs still presents numerous challenges and risks; thus, understanding the advantages and risks of drug combinations may help to better grasp and utilize epi-drugs and offer other perspectives for cancer therapy. In this review, we summarize and discuss the newly developed biomarkers and drug combinations of 12 epi-drugs of the three types of histone modification inhibitors and DNA methylation inhibitors. In any effort to discover and develop small molecule drugs, it has become key to detect drug-likeness and the targeted proteins. For this point, we provide a comprehensive figure to summarize the predicted oral efficacy and target proteins of epi-drugs [15] (Figs. 2, 4). In addition, a large number of natural components function as small-molecule inhibitors. This paper also discusses the progress of natural epi-drugs in recent years to provide a basic reference for clinical trial design and drug compatibility.

## Predictive biomarkers and combination trials of HMT inhibitors

**Fig. 1:**
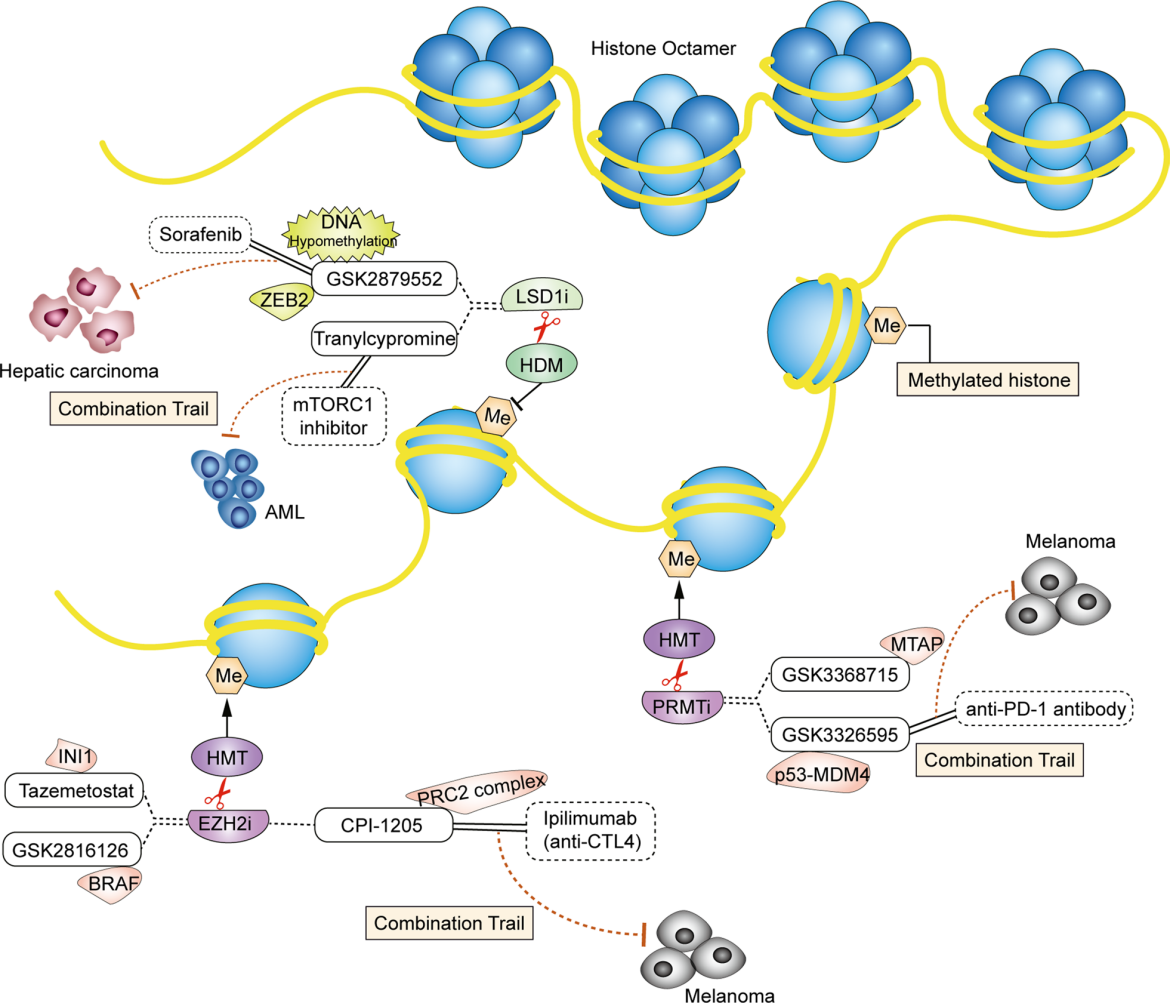
Predictive biomarkers and drug combinations of histone methylation inhibitors. Prominent biomarkers and drug combinations of histone methyltransferase (HMT) and histone demethylase (HDM) inhibitors are detailed here. However, other biomarkers and drug combinations also exist. Histone octamer is the basic unit consisting of the nucleosome core particle. Me represents methylation. HMTs can methylate arginine or lysine of histone, meantime HDMs demethylates methylated histones. The small molecule drugs had been developed to inhibit HMT or HDM to regulate an epigenetic process in cancer cells. The application of these inhibitors acts as anticancer drugs by targeting individual biomarkers through different pathway in a variety of tumors. HMT inhibitors include the PRMT inhibitors GSK3368715 and GSK3326595 and the EZH2 inhibitors tazemetostat, CPI-1205, and GSK2816126. The predicted biomarkers for the two PRMT inhibitors are the MTAP and p53-MDM4 axes, respectively. GSK3326595 combined with immunotherapy can effectively exert synergistic anticancer effects in melanoma. The predicted biomarkers of the three EZH2 inhibitors are INI1, the PRC2 complex, and BRAF. The combination of CPI-1205 and an anti-CTL4 antibody can effectively exert a synergistic anticancer effect in melanoma. LSD1 inhibitors as HDM inhibitors include GSK2879552 and tranylcypromine. The DNA hypomethylation levels and ZEB2 status are predictive biomarkers for the selection of GSK2879552 sensitive patients. In hepatic carcinoma treatment, the combination of GSK2879552 and sorafenib exerts improved anticancer effects. In AML, the combination of tranylcypromine with mTORC1 inhibitors effectively exerts synergistic anticancer effects

**Fig. 2:**
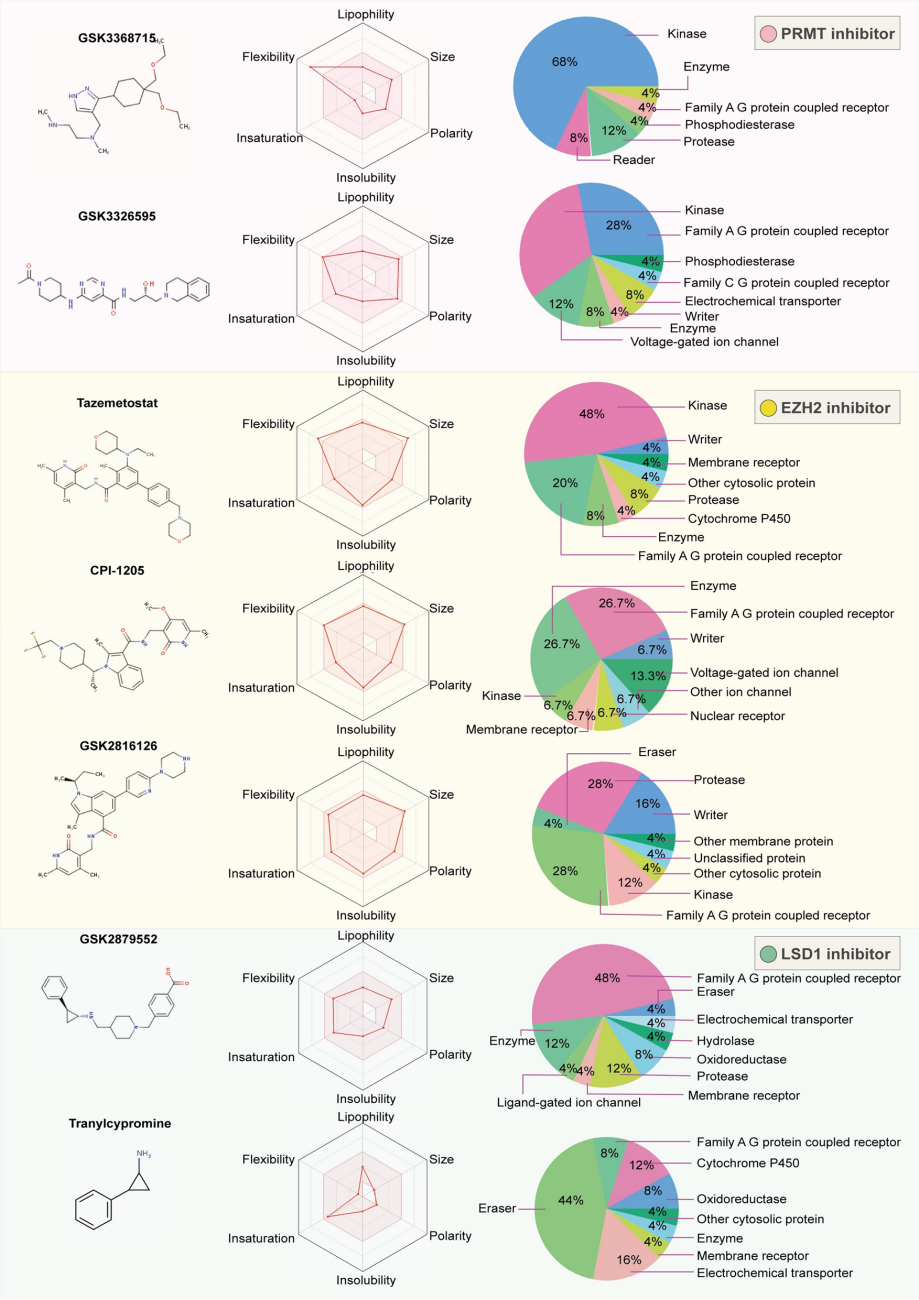
Oral bioavailability and target prediction of PRMT inhibitors, EZH2 inhibitors, and LSD1 inhibitors. We used the online tool SwissADME (https://www.sib.swiss/) to predict the oral bioavailability and target prediction of seven epi-drugs including PRMT inhibitors, EZH2 inhibitors, and LSD1 inhibitors. Oral bioavailability is an essential parameter for determining the efficacy and side effects of new and developing medications. The prediction of oral bioavailability is displayed for a rapid appraisal of drug-likeness. Six physicochemical properties are taken into account: lipophilicity, size, polarity, solubility, flexibility and saturation. The colored zone is the suitable physiochemical space for oral bioavailability. The pink area represents the optimal range for each property. Lipophility: -0.7 < XLOGP3 <  + 5.0; size: 150 g/mol < MV < 500 g/mol; polarity: 20Å2 < TPSA < 130Å2; insolubility: 0 < Log S (ESOL) < 6; insaturation: 0.25 < fraction Csp3 < 1; and flexibility: 0 < Num. rotatable bonds < 9. We also used the website to predict the most probable macromolecular targets of a small molecule, assumed as bioactive of each inhibitor. The pie chart of each inhibitor displays the summary of predication target classes, including kinase, writer, eraser, family A G protein coupled receptor, phosphodiesterase, protease and so on

Histone methylation occurs as a covalent modification of arginine (monomethylation or dimethylation) or lysine (monomethylation, dimethylation, or trimethylation). Histone methylation can participate in cancer-related pathways by inhibiting or activating gene transcription, whereas the abnormal function of HMT affects the occurrence and development of cancer [16–18]. Therefore, the development and study of small-molecule HMT inhibitors opened up new horizons for a novel generation of antineoplastic drugs. HMTs are typically divided into arginine methyltransferase and lysine methyltransferase in humans, such as the PRMT family and EZH2, respectively (Figs. 1, 2). In related reports, high levels of PRMT family proteins and EZH2 expression were found in hematological tumors and solid tumors [19, 20], but the development and application of PRMT inhibitors and EZH2 inhibitors still have limitations.

## PRMT inhibitors

***GSK3368715*** is an oral S-adenosyl-L-methionine (SAM) noncompetitive type 1 PRMT reversible inhibitor. GSK3368715 inhibits the activity of PRMT1, PRMT3, PRMT4, PRMT6, and PRMT8 at different concentrations [21]. The anticancer activity of GSK3368715 was evaluated in more than 200 cell lines of 12 tumor types and in an in vivo model of diffuse large B cell lymphoma (DLBCL) [22]. GSK3368715 treatment showed a promising inhibitory effect in most solid tumor cells, and the tumor inhibition rate of GSK3368715 in renal cell carcinoma, triple-negative breast cancer, and pancreatic cancer was as high as 75%. In addition, researchers also found that the deletion of the methionine phosphorylase (MTAP) gene leads to the accumulation of metabolite 2-methyladenosine in cells, which enhances the sensitivity of certain tumor cells to GSK3368715. Therefore, the MTAP status provides a theoretical basis as a biomarker for patients treated with GSK3368715 [23]. In terms of clinical trials, the first human study of GSK3368715 in solid tumors, including pancreatic cancer, bladder cancer, non-small cell lung cancer, and recurrent/refractory DLBCL, is currently under recruitment (NCT03666988) (Table 1). This study will evaluate the safety, pharmacokinetics, pharmacodynamics, food effects, and preliminary clinical activity of GSK3368715.

**Table 1:** Clinical trials and condition of PRMT, EZH2, LSD1, HDAC and DNMT1 inhibitors

Category	Drug	NCT number	Study title	Condition	Status	Phase
PRMT inhibitors	GSK3368715	NCT03666988	First Time in Humans (FTIH) Study of GSK3368715 in Participants with Solid Tumors and DLBCL	Neoplasms	Recruiting	Phase 1
	GSK3326595	NCT02783300	Dose Escalation Study of GSK3326595 in Participants With Solid Tumors and Non-Hodgkin's Lymphoma (NHL)	Neoplasms	Recruiting	Phase 1
		NCT03614728	Study to Investigate the Safety and Clinical Activity of GSK3326595 and Other Agents to Treat Myelodysplastic Syndrome (MDS) and AML	Neoplasms	Recruiting	Phase 1
EZH2 inhibitors	Tazemetostat	NCT02860286	Study of the EZH2 Inhibitor Tazemetostat in Malignant Mesothelioma	Mesothelioma, BAP1 Loss of Function	Completed	Phase 2
		NCT02220842	A Safety and Pharmacology Study of Atezolizumab (MPDL3280A) Administered With Obinutuzumab or Tazemetostat in Participants With Relapsed/Refractory Follicular Lymphoma and DLBCL	Lymphoma	Completed	Phase 1
		NCT03010982	Open-Label, Multi-Center, Two-Part, Ph1 Study to Characterize the PKs of an Intravenous Micro-Dose of [14C]-Tazemetostat (EPZ 6438) and the ADME of an Oral [14C]-Labeled Dose of Tazemetostat in Subjects With B Cell Lymphomas or Adv Solid Tumors	Several Types of Lymphoma, Advanced Solid Tumors	Completed	Phase 1
	CPI-1205	NCT02395601	A Study Evaluating CPI-1205 in Patients With B Cell Lymphomas	B Cell Lymphoma	Completed	Phase 1
	GSK2816126	NCT02082977	A Study to Investigate the Safety, Pharmacokinetics, Pharmacodynamics and Clinical Activity of GSK2816126 in Subjects With Relapsed/Refractory DLBCL, Transformed Follicular Lymphoma, Other NHLs, Solid Tumors and MM	Cancer, Neoplasms	Terminated	Phase 1
LSD1 inhibitors	GSK2879552	NCT02034123	Investigation of GSK2879552 in Subjects With Relapsed/Refractory Small Cell Lung Carcinoma (SCLC)	SCLC	Terminated	Phase 1
		NCT02929498	Safety, Clinical Activity, Pharmacokinetics (PK) and Pharmacodynamics Study of GSK2879552, Alone or With Azacitidine, in Subjects With High Risk MDS	MDS	Terminated	Phase 1/2
		NCT02177812	A Phase I Dose Escalation Study of GSK2879552 in Subjects With AML	AML	Terminated	Phase 1
	Tranylcypromine (TCP)	NCT02273102	Study of TCP-ATRA for Adult Patients With AML and MDS	AML, MDS	Completed	Phase 1
		NCT02261779	Phase I/II Trial of ATRA and TCP in Patients With Relapsed or Refractory AML and no Intensive Treatment is Possible	AML	Unknown	Phase 1/2
		NCT02717884	Study of Sensitization of Non-M3 AML Blasts to ATRA by Epigenetic Treatment With TCP	AML, MDS	Recruiting	Phase 1/2
HDAC inhibitors	Vorinostat	NCT00918489	Study on Efficacy and Tolerability of Virinostat in Patients With Advanced, Metastatic Soft Tissue Sarcoma (STS) (SAHA-I)	STS	Completed	Phase 2
		NCT00735826	A Clinical Trial to Validate Molecular Targets of Virinostat in Patients With Aerodigestive Tract Cancer	Aerodigestive Tract Cancer, Lung Cancer, Esophageal Cancer, Head and Neck Cancer	Completed	Not Applicable
	Abexinostat	NCT03592472	A Study of Pazopanib With or Without Abexinostat in Patients With Locally Advanced or Metastatic Renal Cell Carcinoma (RENAVIV)	Renal Cell Carcinoma	Active, not recruiting	Phase 3
	Panobinostat	NCT01336842	Study of Cisplatin and Pemetrexed in Combination With Panobinostat in Solid Tumors	Solid Tumors, NSCLC	Completed	Phase 1
DNMT1 inhibitors	5'-Azacytidine (azacytidine)	NCT02940483	Infusion of 5-Azacytidine (5-AZA) Into the Fourth Ventricle in Children With Recurrent Posterior Fossa Ependymoma (5-AZA)	Brain Tumor Recurrent	Completed	Early Phase 1
		NCT02993523	A Study of Venetoclax in Combination With Azacitidine Versus Azacitidine in Treatment Naïve Subjects With AML Who Are Ineligible for Standard Induction Therapy	AML	Active, not recruiting	Phase 3
	Decitabine	NCT02957968	Neoadjuvant Pembrolizumab + Decitabine Followed by Std Neoadj Chemo for Locally Advanced HER2- Breast Ca	Breast Cancer	Recruiting	Phase 2
		NCT04252248	Decitabine Treatment in HPV-Induced Anogenital and Head and Neck Cancer Patients After Radiotherapy or as Novel Late Salvage	Head and Neck Cancer, Anogenital Cancer	Recruiting	Phase 1
		NCT02961101	Anti-PD-1 Antibody Alone or in Combination With Decitabine/Chemotherapy in Relapsed or Refractory Malignancies	Multiple Malignancies	Recruiting	Phase 1/2

***GSK3326595*** is a selective reversible inhibitor of PRMT5 under clinical development. In breast cancer and lymphoma cell lines, GSK3326595 activates the p53 pathway by inducing alternative splicing of murine double minute 4 (MDM4), regulating the cell cycle to stimulate apoptosis [24]. The p53 protein is classified as a tumor suppressor, and the p53 pathway can be interacted with many other transduction pathways such as Wnt/β catenin, IGF-1/AKT or p38 MAPK [25]. Thus, the integrity of the p53-MDM4 axis in patients may be a potential biomarker for the clinical administration of GSK3326595 [26]. Another small-molecule inhibitor, the cyclin-dependent kinase 4/6 (CDK4/6) inhibitor palbociclib, has been widely studied in the treatment of melanoma. However, there are still certain melanoma cells with acquired drug resistance to palbociclib. In the development of combination trials, it was found that the combination of palbociclib and GSK3326595 could alter the pre-mRNA splicing of MDM4, attenuating the expression of MDM4 to activate p53, further leading to the inhibition of CDK2, eventually resulting in the loss of the acquired drug resistance of palbociclib [27]. Moreover, during the application of GSK3326595 against melanoma, GSK3326595 treatment augmented the level of MHC1 and had a significant inhibitory effect on melanoma in immunoactivity mice. The combination of GSK3326595 and immunotherapy (with an anti-PD-1 antibody) effectively controlled the growth of melanoma and improved the therapeutic effect of a single drug [24]. Current clinical trials on GSK3326595 recruiting patients include a study of the safety and clinical activity of GSK3326595 in the treatment of acute myeloid leukemia (AML) (NCT03614728) and a study of the clinical activity and dose progression of oral GSK3326595 in selected solid tumors and non-Hodgkin's lymphoma (NCT02783300) (Table 1).

## EZH2 inhibitors

***Tazemetostat*** is an oral, SAM competitive EZH2 inhibitor, which selectively inhibits the activity of wild-type and mutant EZH2 [28]. The United States Food and Drug Administration (FDA) has approved tazemetostat as the first epi-drug for sarcoma in adults and children over the age of 16 with metastatic or locally advanced epithelioid sarcoma who are not suitable for radical surgery in 2020 [29–31]. In early research, tazemetostat has demonstrated anticancer efficacy in a variety of cancers with functional deletion mutations of the SWI/SNF complex or abnormal activation of EZH2 resulting in histone hypermethylation, such as sarcoma, non-Hodgkin's lymphoma, medulloblastoma, and many solid tumors [32]. As a component of the SWI/SNF complex, INI1 is a powerful tumor suppressor gene. In solid tumors with loss of INI1 function, EZH2 can be abnormally recruited, leading to the activation of multiple oncogenes signaling pathways. Therefore, in vivo and in vitro studies have revealed that treatment with tazemetostat can effectively prevent the proliferation and survival of INI1-negative malignant rhabdoid tumor cells [33, 34]. Tazemetostat plays an active role in different types of tumors. In medulloblastoma, tazemetostat reactivates the expression of BAI1 by regulating EZH2 levels, thus preventing the growth of medulloblastoma cells and prolonging the survival time of the orthotopic xenotransplantation model [32]. There are 23 tazemetostat-related clinical trials. Among them, nine are under recruitment, whereas three trials on combination therapy for lymphoma, solid tumors, and malignant stromal tumors have been completed (NCT03010982, NCT02220842, and NCT02860286) (Table 1). Among the different tumor types that depend on the abnormal growth of EZH2, a better study of tazemetostat administration based on biomarkers may be helpful for clinical drug usage.

***CPI-1205***, an oral indole EZH2 inhibitor, inhibits tumor growth in B cell lymphoma and several types of solid tumors [35, 36]. The PRC2 complex is involved in the process of histone methylation, and its dysfunction is related to the occurrence and development of malignant tumors and tumor prognosis [37–39]. The eutectic structure of the CPI-1205 and PRC2 complex plays an important role in anti-tumor effects [40]. In addition, immunotherapy combined with CPI-1205 treatment exerts an improved anticancer function via the modification of EZH2 expression in melanoma. CPI-1205 administration suppresses EZH2 to trigger the phenotype of Tregs in human T cells, whereas the treatment with ipilimumab (an anti-CTLA-4 monoclonal antibody) augments the expression of EZH2 in peripheral blood T cells. Thus, the combination of CPI-1205 and ipilimumab alleviates the side effects of ipilimumab monotherapy, providing a basis for the targeting of the combination therapy [41]. Currently, there are three clinical trials of CPI-1205, including a phase I clinical trial to evaluate the efficacy of CPI-1205 in B cell lymphoma (NCT02395601) (Table 1). These results provide a credible basis for CPI-1205 as a potential cancer treatment.

***GSK2816126*** inhibits wildtype and mutant EZH2 by competing with SAM as an effective EZH2 inhibitor. GSK2816126 exhibits outstanding anticancer ability in EZH2 mutated malignant tumors and can be administered intravenously in preclinical experiments [42]. In addition, BRAF abnormalities are also present in EZH2 mutated cancers. The combination of the BRAF inhibitors vemurafenib and GSK2816126 revealed a more significant anticancer effect than vemurafenib monotherapy in melanoma models with both a BRAF V600E mutation and an EZH2 abnormality [43]. Therefore, the status of the BRAF V600E mutation with an EZH2 gene copy number variation can be used as a potential tumor therapeutic target. Phase I clinical trials of GSK2816126 in patients with lymphoma and solid tumors have been completed. GSK2816126 was highly active in inhibiting EZH2 mutated tumor growth with a maximum tolerated dose of 2400 mg by intravenous infusion and a dose-limiting toxicity of hepatic transaminitis (NCT02082977) (Table 1) [44].

## Predictive biomarkers and combination trials of HDM inhibitors

Histone methylation occurs at lysine and arginine sites and is a reversible process that can be demethylated by HDMs, mainly by LSD1 and demethylases of the JmjC family [45, 46]. As an important mechanism of epigenetic modification, histone demethylation may be involved in the regulation of chromatin remodeling, embryonic development, cellular senescence, tumor proliferation, survival, and other key biological processes [47–49]. The first identified HDM LSD1 is a flavin adenine dinucleotide-dependent monoamine oxidase that can be involved in transcriptional regulation. LSD1 has become a promising epigenetic target for a variety of malignant tumors, and its function mainly depends on the demethylase activity at the C terminus. Inhibition of LSD1 activity disturbs the invasion, proliferation, and survival of cancer cells [50, 51]. Therefore, LSD1 inhibitors developed for this mechanism are currently widely used in anti-tumor research and combined immunotherapy (Fig. 1).

## LSDI inhibitors

***GSK2879552*** is an oral, irreversible cyclopropylamine LSD1 inhibitor that is used as an effective antineoplastic drug in a variety of tumors. The antitumor effect of GSK2879552 was examined in 165 cancer cell types, indicating a significant inhibitory effect on 30% of small cell lung cancers and AML. In vitro and in vivo experiments on sensitive small cell lung cancers showed that the MYCL1 copy number, DNA hypomethylation, and TGFβ pathway are associated with GSK2879552 sensitivity to growth inhibition. Helai P. Mohammad identified 45 differentially methylated probes distributed promoter, intronic, and intergenic regions; hence, SMAD2 binding sequences were enriched at differentially methylated regions in sensitivity to GSK2879552. Therefore, the hallmarks of predicted DNA hypomethylation could serve as biomarkers for tumors sensitive to GSK2879552 [52]. In AML, the GSK2879552 therapeutic strategy extinguishes the interaction of ZEB2-KDM1A targeting cells with high ZEB2 levels, inhibits tumor invasion and growth, and then affects tumor survival. The ZEB2 status may act as a biomarker to cope with hematological or solid tumors driven by ZEB2 for GSK2879552 treatment [53]. GSK2879552 also exerts efficacy in combination with other classical antineoplastic drugs and small-molecule inhibitors against cancer. The combination of GSK2879552 with all-trans retinoic acid is also considered in AML, exhibiting a better synergistic effect on cell proliferation and cytotoxicity, ultimately resulting in caspase-mediated cell death, which may achieve ideal anticancer effects in relapsed and refractory AML patients [54]. Resistance often occurs with the tyrosine kinase inhibitor sorafenib in advanced liver cancer patients, but GSK2879552 and sorafenib mechanistically impede the Wnt/β-catenin pathway to attenuate cancer cell stemness, inhibiting acquired resistance and improving the therapeutic effect of sorafenib [55]. Currently, there are three terminated clinical trials associated with GSK2879552 (Table 1). Among them, a phase I trial of GSK2879552 in small cell lung cancer was terminated, because the disease control rate in patients was too high, although the pharmacokinetic properties of the drug were good (NCT02034123).

***Tranylcypromine (TCP)*** was originally used in clinical applications as an antidepressant targeting monoamine oxidase A and B, which are related to the structure of LSD1. Moreover, a TCP analog has been widely recognized as an effective reversible LSD1 inhibitor [56]. In the treatment of AML, a TCP analog effectively inhibited LSD1 interference with GFI1-mediated PU1 target gene inhibition and induced AML differentiation as an LSD1 inhibitor [57]. Since a combination therapy can provide better clinical efficacy than TCP treatment alone, there are a number of achievements in the research of TCP-related combinations for AML therapy. Multiple components of the mTORC1 signaling act as sensitizers for LSD1 inhibition in AML. Among them, the combination of MTORC1 components or mTORC1 inhibitors with TCP, which promotes the differentiation of cell lines and primary cells and enhances MLL-translocated AML differentiation, can be evaluated in early clinical trials [58]. Corin, a synthetic hybrid derived from the HDAC inhibitors entinostat and a TCP analog, showed strong anticancer activity by comprehensively blocking the CoREST complex in melanoma and skin scale-cell carcinoma. Such two-pronged hybrids demonstrate the preferential targeting of specific epigenetic regulatory the CoREST complex and provide unique therapeutic opportunities [59]. There are 26 clinical trials of TCP, and three tumor-related therapeutic trials mainly focus on AML treatment (NCT02261779, NCT02717884, NCT02273102) (Table 1).

## Predictive biomarkers and combination trials of HDAC inhibitors

**Fig. 3:**
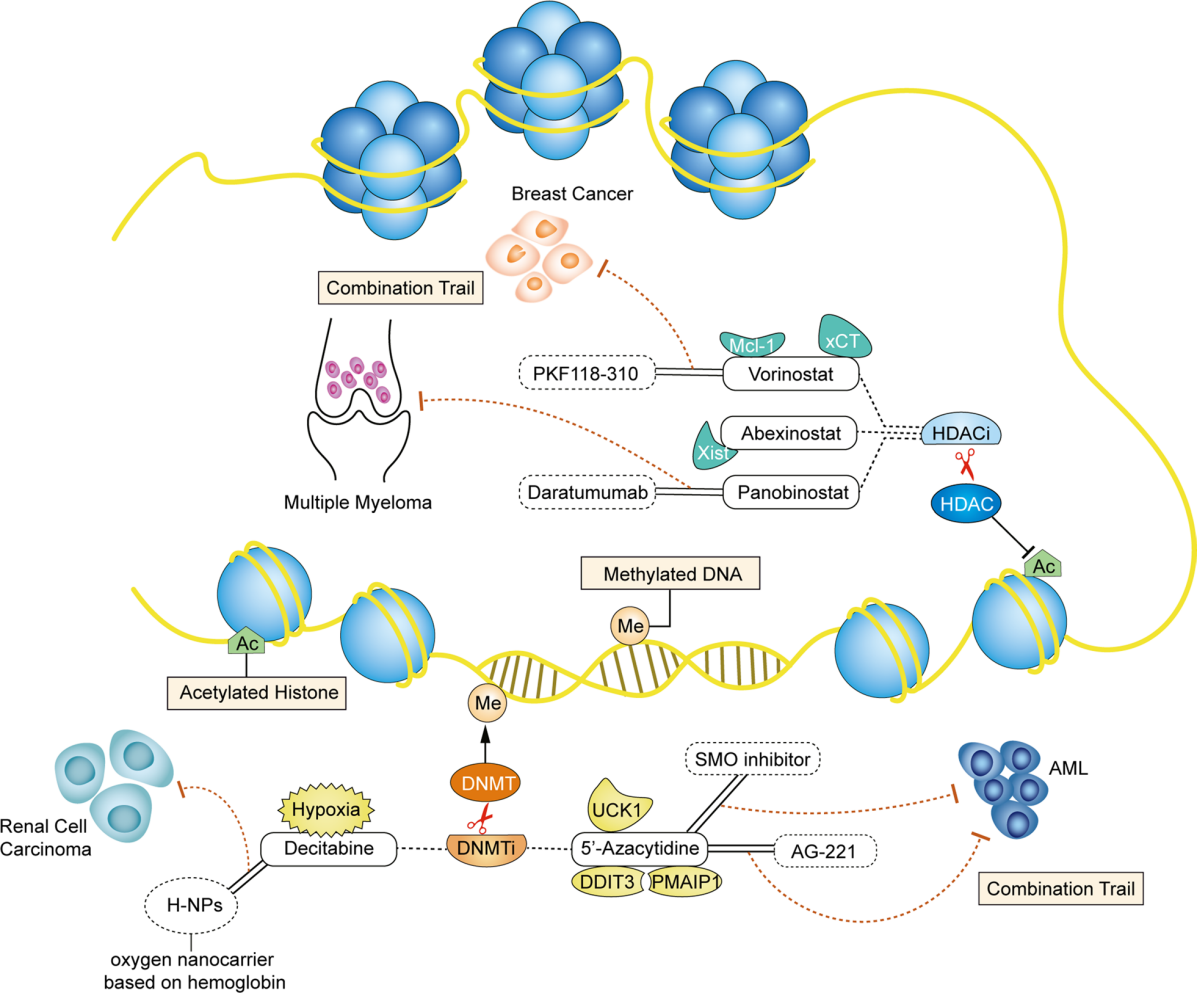
Predictive biomarkers and drug combinations of histone deacetylation (HDAC) inhibitors and DNA methyltransferase (DNMT) inhibitors. Prominent biomarkers and drug combinations of HDAC/DNMT inhibitors are detailed here. However, other biomarkers and HDAC/DNMT inhibitor combinations also exist. Me represents methylation. Ac represents acetylation. Similar to histone methylation, methylation of DNA occurs at cytosine residues and needs the participation of DNMT. Besides, histone acetylation has consistently been linked to a chromatin state and regulated by histone acetylases (HATs) and HDACs. The HDAC inhibitors include vorinostat, abexinostat, and panobinostat. The predicted biomarkers for the three HDAC inhibitors are Mcl-1, xCT, and *Xist*. Vorinostat combined with PFK 118–310 can effectively exert synergistic anticancer effects in breast cancer. Further, panobinostat combined with daratumumab can effectively exert synergistic anticancer effects in multiply myeloma. The DNMT inhibitors include 5'-azacytidine and decitabine. The 5'-azacytidine predicted biomarkers are the UCK1, DDIT3, and PMAIP1 status. The combination of 5'-azacytidine with a SMO inhibitor or AG-221 exerts improved anticancer effects in acute myeloid leukemia. Cellular hypoxia is a predictive biomarker for the selection of decitabine sensitive patients. In renal cell carcinoma, the combination between decitabine and the oxygen nanocarrier H-NPs exerts synergistic anticancer effects

**Fig. 4:**
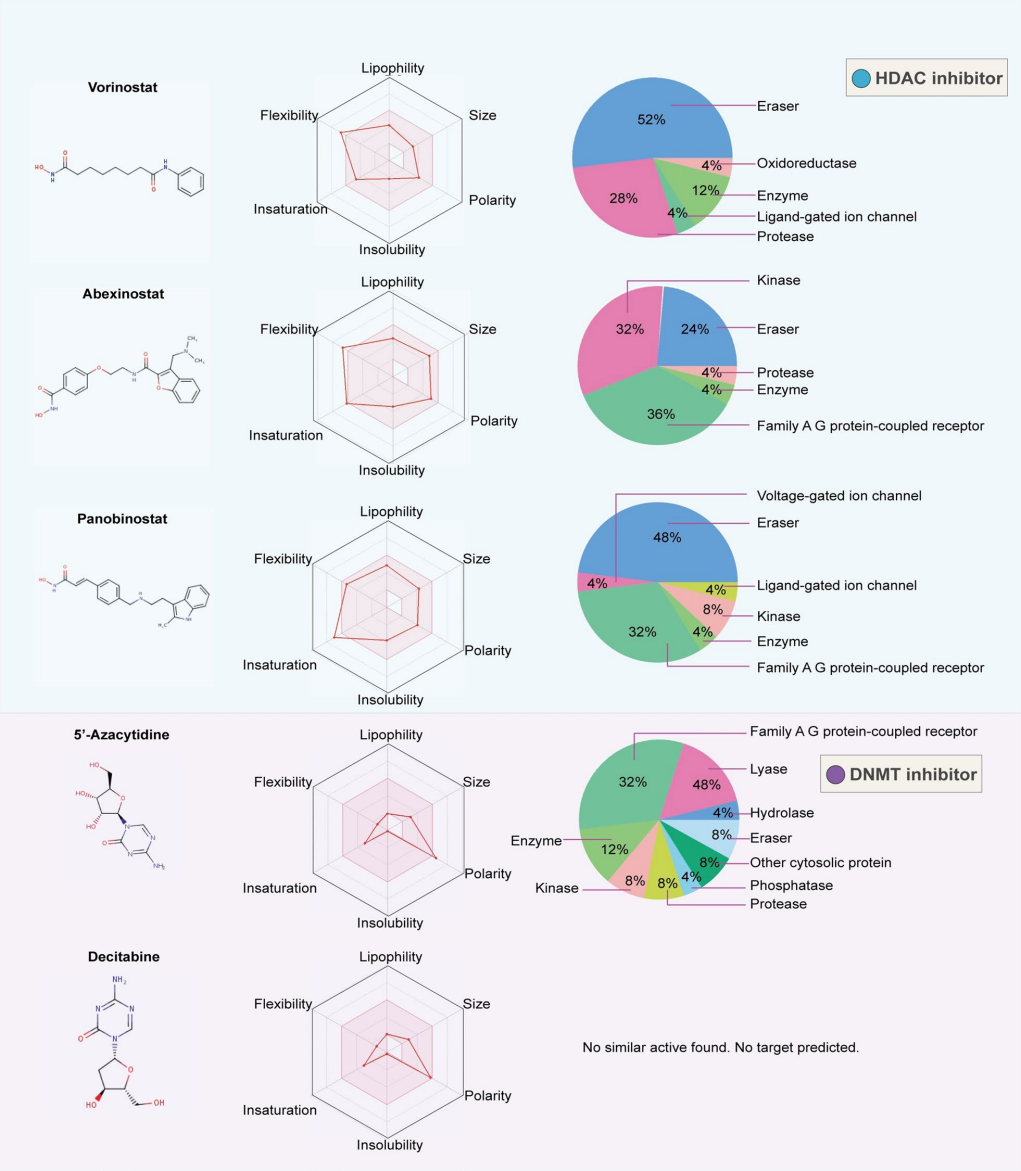
Oral bioavailability and target prediction of HDAC inhibitors and DNMT inhibitors. We used the online tool SwissADME (https://www.sib.swiss/) to predict the oral bioavailability and target prediction of 5 epi-drugs that represent HDAC inhibitors or DNMT inhibitors. The colored zone is the suitable physiochemical space for oral bioavailability. The pink area represents the optimal range for each property. Lipophility: -0.7 < XLOGP3 <  + 5.0; size: 150 g/mol < MV < 500 g/mol; polarity: 20Å^2^ < TPSA < 130Å^2^; insolubility: 0 < log S (ESOL) < 6; insaturation: 0.25 < fraction Csp3 < 1; flexibility: 0 < num. rotatable bonds < 9. The pie chart of each inhibitor displays the summary of predication target classes

Acetylation occurs on specific lysines of four histones, and the involvement of two key enzymes in the process is required to be catalyzed by a HAT and an HDAC. The HAT acetylates histone lysine residues to activate gene transcription, whereas the HDAC acts as a protease to inhibit gene transcription by deacetylating acetylated histones [60–62]. In cancer cells, the overexpression of HDAC enhances histone deacetylation and tightens loose nucleosomes to constrain the expression of tumor suppressor genes, thereby affecting the proliferation, metastasis, and survival of cancer cells. Therefore, HDAC inhibitors promote histone acetylation, relax nucleosomes, and activate the transcription of genes to induce programmed cell death, such as apoptosis [63–65]. This class of small-molecule inhibitors has thus become a new class of anti-tumor drugs, but some clinical trials exhibit numerous side effects of pan-HDAC inhibitors, such as hematologic toxicity, diarrhea, weight loss, taste disturbances, electrolyte changes, disordered clotting, fatigue, and cardiac arrhythmias [66]. These findings are not surprising if only considers the essential role of HDACs as key regulators of genes transcription in any tumor type without considering the distinctive HDAC family members and the characteristic of different tumors. In order to achieve good therapeutic effects on a variety of malignant tumors with low toxicity, and high efficiency, we have to cogitate the selectivity of HDAC inhibitors in diverse cancer types. The following paragraphs focus on biomarker and combination guidance for three FDA-approved HDAC inhibitors, vorinostat, abexinostat, and panobinostat (Figs. 3, 4).

***Vorinostat*** (also called SAHA), an oral HDAC inhibitor, can effectively inhibit the activity of HDAC1, HDAC2, HDAC3, and HDAC6, stimulating stem cell differentiation to affect cell cycle and cell death, and has been approved by the FDA for the treatment of cutaneous T cell lymphoma [67, 68]. Vorinostat exerts antitumor activity through different targeted regulatory mechanisms, such as the induction of BCL-2-induced apoptosis, reduction of glycolysis in a c-Myc-dependent manner to inhibit ATP levels, or synergistic involvement in redox pathways to induce ferroptosis [69]. The treatment of squamous carcinoma cells with vorinostat regulates the expression of Bcl-2 family members and suppresses Mcl-1, a major and tissue-specific survival factor in squamous cell carcinoma, thereby inducing apoptosis. In addition, FBW7-mutant somatic cells are more sensitive to vorinostat treatment. The Mcl-1 status and FBTW7 mutations may act as biomarkers for the application of vorinostat as the basis for the sensitivity of certain tumors to HDAC inhibitor therapy [70, 71]. Moreover, oxidative stress status is also involved in the directive of vorinostat sensitivity; glutamate-cystine transporter xCT levels present a positive correlation with vorinostat in a variety of cancer cells. When vorinostat is combined with the xCT inhibitor SASP, it causes ROS accumulation and induces ferroptosis; thus, xCT levels are also expected to be a potential predictive biomarker for vorinostat treatment [72, 73]. The combination of vorinostat with the natural component artemisinin succinate (ARS) increases the expression of 5-aminolevulinic acid synthase (ALAS1) and enhances the cytotoxicity of ARS by regulating heme synthesis, which has a synergistic antitumor effect and is more effective than monotherapy in solid tumors [74]. In breast cancer, the combined application of vorinostat and the Wnt-β-linked protein blocker PKF118-310 facilitates the induction of differentiation of cancer stem cells to inhibit the EMT process, reducing the number of breast cancer stem cells through a nanoparticle delivery system [75].

***Abexinostat***, as a moderate oral, isotaximate-containing HDAC inhibitor, can effectively inhibit the activity of HDAC1, HDAC2, HDAC3, HDAC6, HDAC8, and HDAC10. Moreover, abexinostat has been approved by the FDA for the treatment of fourth-line follicular lymphoma as a non-cell cycle-specific cytotoxic epi-drug [76]. Abexinostat appears to have good clinical efficacy in hematological and solid tumors and exhibits better safety than other HDAC inhibitors of the same class. Abexinostat, due to its prominence for DNA double-strand break repair and homologous recombination (HR), plays an anticancer role by decreasing the expression of HR-related gene RAD15, and reducing the ability for HR repair [77]. Interestingly, we demonstrated that abexinostat can participate in the calcium signaling pathway in breast cancer. Abexinostat dysregulated cellular calcium influx via inhibiting Gαq-PLCβ3-mediated calcium signaling by activating the transcription of RGS2, leading to the inhibition of cell proliferation and EMT progression, eventually resulting in cell death in breast cancer [78]. Abexinostat is an effective anticancer drug for treatment-resistant gallbladder cancer, which suppresses cellular growth and proliferation by inhibiting ErbB2 levels and downregulating miR-21 expression [79]. Abexinostat could meritoriously induce the differentiation of breast cancer stem cells with low expression of the long non-coding RNA *Xist*, increasing the sensitivity to drug response to further induce apoptosis in a variety of breast cancer cell lines. Therefore, *Xist* may be a predictive biomarker in abexinostat sensitive patients for the exertion of abexinostat anti-tumor characteristics for accurate treatment [80]. Abexinostat has obtained great research value in combination therapy. In addition, the combination of abexinostat with bortezomib displays a good synergistic effect in lymphoma, and the combination of abexinostat with chemotherapy also significantly inhibits tumor growth and metastasis in soft tissue sarcoma; thus, these combination trials deserve further rigorous evaluation in clinical studies [81, 82].

***Panobinostat*** is an oral, nonselective pan-HDAC inhibitor that exerts severe stress on cancer cells to trigger cell death, whereas healthy cells are unaffected. Therefore, panobinostat was approved by the FDA for drug combination with bortezomib or dexamethasone as a third-line treatment in patients with multiple myeloma (MM) [83–85]. It should be noted that panobinostat has a black box warning, indicating that the drug has adverse side effects accompanied by severe diarrhea, fatal cardiac events, arrhythmias, and electrocardiogram (ECG) changes. Likewise, panobinostat is usually considered for combination therapy with other agents to treat malignancies. Panobinostat combined with the anti-CD38 monoclonal antibody danatuximab augments CD38 expression and enhances danatuximab monotherapy with significant anti-tumor activity in MM [86]. MEK inhibition has revealed surprising anti-tumor effect in BRAF-mutant melanoma. RAF and MEK inhibition can improve the MAPK signaling and adapt AKT signaling [87, 88]. Surprisingly, the combination of MEK inhibitors with panobinostat is even more effective than other drug combinations by inhibiting the PI3K/AKT pathway and ET-3-mediated YAP signaling, which in turn effectively inhibits tumor growth in subcutaneous and liver metastasis models [89]. Thus, among many HDAC inhibitors, panobinostat has a high-intensity anticancer effect as a combination drug.

## Predictive biomarkers and combination trials of DNMT1 inhibitors

DNA methylation is catalyzed by DNMT to obtain a chemical modification with a methyl group by covalent bonding [90]. This modification process does not change the DNA sequence but can induce chromatin structure, DNA stability, and DNA and protein interaction changes to participate in the regulation of gene expression, embryonic development, and control of cellular function and tumorigenesis [91]. DNMT is divided into three families in mammals: DNMT1, DNMT2, and DNMT3. DNMT1, the most widely studied enzyme in the DNMT family, is a key enzyme in DNA replication repair and methylation maintenance [92, 93]. DNMT1 inhibitors are currently widely studied as epigenetic drugs for cancer treatment, and two DNMT inhibitors, azacytidine and decitabine, have been approved by the FDA for certain cancer treatments (Figs. 3, 4).

***Decitabine***, an irreversible DNMT1 inhibitor, is also a deoxycytidine analog, antimetabolite, which mainly affects the cell cycle and promotes apoptosis. Decitabine behaves as a suicide substrate for DNA methyltransferases and incorporation into DNA without the requirement of a deoxygenation step [94]. Decitabine is approved by the FDA for the treatment of myelodysplastic syndrome. It is worth noting that the anticancer activity of decitabine has a dual mechanism of dose difference, with cytotoxic effects at high concentrations and demethylation effects at low concentrations, indicating a bigger role in the clinical application of malignant tumors. In small cell renal cancer, decitabine promotes T cell activation, enhancing the cancer cell response to immune checkpoint blockade and immune infiltration by stimulating the expression of the transposable element ERV and cytokine secretion [95]. Organic cation/carnitine transporter 2 (OCT2) acts as an important protein involved in renal excretion, and the loss of OCT2 in renal cell carcinoma may lead to decitabine resistance [96, 97]. By combining decitabine and the hemoglobin-based oxygen nanocarrier H-NPs to regulate the hypoxic properties of renal cancer cells, the loss of decitabine activity can be alleviated, enhancing the OCT2 transcriptional process to increase the sensitivity of cancer cells to decitabine treatment. Therefore, modulating the cellular hypoxic environment upon decitabine treatment may serve as a potential clinical application guide for modulating drug resistance [98]. There are more than 400 clinical trials for decitabine, of which 159 have been completed and 110 are recruiting, mainly focusing on hematological oncology and drug combinations, and only a few solid tumor studies of breast, head, and neck cancer (NCT02957968, NCT04252248, and NCT02961101) (Table 1).

***5'-Azacytidine (azacytidine),*** a pyrimidine nucleoside analog, can inhibit DNMT1 activity, weaken DNA methylation, and reverse epigenetic changes. Azacytidine binds to RNA as an antimetabolite of cytidine and has been approved by the FDA for preleukemic myelodysplastic syndrome [99]. Unlike the action mechanism of decitabine, azacytidine is predominantly incorporated into RNA rather than DNA. DNMT displays the nucleophilic attack by the dynamic site Cys residue and methylation by SAM [100]. A recent study on the resistance to azacytidine in AML revealed that azacytidine treatment regulates uridine-cytidine kinase 1 (UCK1) ubiquitination and phosphorylation around the KLHL2/USP28/ATM axis, affecting cellular proliferation and apoptosis. Moreover, the prediction of low levels of UCK1 may be an important candidate biomarker for azacytidine efficacy [101]. In addition, azacytidine activates the integrated stress response (ISR) pathway to induce the activated expression of DDIT3, the classical target in AML, and PMAIP1 and BBC3. Thus, the combination treatment of azacytidine with venetoclax in patients with PMAIP1 and DDIT3 status as potential biomarkers is currently undergoing a phase III clinical trial (NCT02993523) (Table 1) [102]. When azacytidine is used as an anticancer drug, the combination of targeted signaling pathways and epigenetic pathways can effectively improve drug resistance and enhance sensitivity [103]. TET2 and IDH2 mutations resulting from abnormal DNA methylation often occur in AML. Moreover, after the combination of azacytidine with the IDH2 inhibitor AG-221, it can alleviate abnormal changes in DNA methylation, causing a decrease in the number of leukemia cells, which in turn is consistent with anti-leukemia activity to enhance sensitivity [104–106]. In elderly patients with AML, an increased rate of drug response to azacytidine treatment is observed. Nonetheless, the sensitizing potential of azacytidine in elderly patients is not associated with common myeloid mutations. Genetic targets in the common deletion region of chromosomes 5 and 7 affect azacytidine sensitivity in elderly patients, such as the silencing of SMO. Consequently, SMO inhibitors combined with azacytidine effectively curb the hedgehog pathway and improve the anti-tumor activity of azacytidine [107].

## Natural components as epi-drugs

Natural drugs originate from pharmacologically active ingredients in animals, plants, or minerals, such as international hot natural drugs, the anticancer drug paclitaxel and its derivatives, and the antimalarial drug artemisinin [108]. Due to concerns about the negative effects of chemicals on health and life, the research and development of natural ingredients as drugs have been vigorously carried out in recent years. Further, ordinances on natural drugs have started to relax, and the USA has been unwinding the restrictions on botanicals by modifying the relevant provisions of the FDA. Gradually, natural components have been more intensively studied by researchers. Although the mechanism by which epi-drugs exert epigenetic modifications as enzyme inhibitors has been frequently reported, no drugs have been developed for clinical use. The natural phytoconstituents resveratrol and curcumin act as DNMT inhibitors to exert anticancer effects in certain solid tumors by inhibiting the activity and expression of DNMT and the hypermethylated tumor suppressor gene RASSF1A [109–112]. There are many related studies on natural inhibitors of histone-modifying enzymes. For example, curcumin, sulforaphane, tanshindiols, rottlerin, and other plant extracts exhibit EZH2 inhibitory activity, which in turn inhibits histone methylation and attenuates tumor proliferation and invasion to induce cellular apoptosis [113–116]. The anticancer potency of olive oil in MM was investigated; olive oil inhibited the expression of HDAC1, HDAC2, HDAC3, HDAC4, and HDAC6; arrested the cell cycle; and induced caspase 8-dependent apoptosis by regulating Sp1 without affecting the DNMT activity [117]. Moreover, the extracts of natural products also can act as the epigenetic regulator, such as ibotenic acid extracted from fungal species and baicalein exhibit HDAC inhibitory activity to impede the progression of cancer [118, 119] (Table 2). Lycopene, a potent antioxidant, extracted from tomatoes, carrot, watermelon has been identified as a potential DNA methylating agent to reduce genomic instability in breast cancers [120, 121]. As a natural, easy obtainment, high value and potential in use plant pigment, lycopene appears epigenetic activity in various types of cancer. Thus, the in-depth epigenetic mechanism of lycopene is worthy of further excavation and exploration.

**Table 2:** Natural product of the epigenetic target and clinical trials

Natural product	Epigenetic target	Condition	References	Clinical trial (Condition)
Resveratrol	DNMT	Breast cancer	95	NCT00256334 (Colon Cancer) NCT00433576 (Colon Cancer)
Curcumin	DNMT EZH2	Prostate cancer Lung cancer	96–99	NCT03211104 (Prostate cancer) NCT01333917 (Colon Cancer) NCT02439385 (Colon Cancer) NCT01160302 (Head and Neck Cancer) NCT01042938 (Breast cancer) NCT00113841 (Multiple Myeloma)
Sulforaphane	EZH2	Melanoma	100	NCT01228084 (Prostate cancer) NCT00946309 (Prostate cancer) NCT00894712 (Breast cancer) NCT00982319 (Breast cancer)
Tanshindiols	EZH2	B cell lymphoma	101	No studies
Rottlerin	EZH2	Prostate cancer	102	No studies
Olive oil	HDAC	Multiple Myeloma	103	NCT02599103 (Colorectal Neoplasms)
Ibotenic acid	HDAC7	Breast cancer	104	No studies
Baicalein	HDAC	Core binding factor-acute myeloid leukemia	105	No studies

Although the development of natural epi-drugs has opened new avenues to reduce the side effects of cancer therapy, the target diversity of natural drug components poses great difficulties for clinical medication guidance. For example, curcumin has the efficacy of EZH2 and DNMT inhibitors. Thus, the pathway of curcumin’s anti-tumor activity is complex, and predicting its biomarker localization in suitable patients is difficult. Moreover, there are too many kinds of natural components, and the systematic screening of natural drugs for their inhibitory effects on epigenetic enzymes still faces great challenges. Even if many natural ingredients with suspected epi-drug properties are screened out, the comprehensive mechanistic interpretation of natural ingredients requires tremendous energy. Still, as long as the road to cancer treatment continues, the pace of research on natural epi-drugs with low side effects will not stop. Future clinical applications for natural drugs are awaited.

## Perspectives

**Fig. 5:**
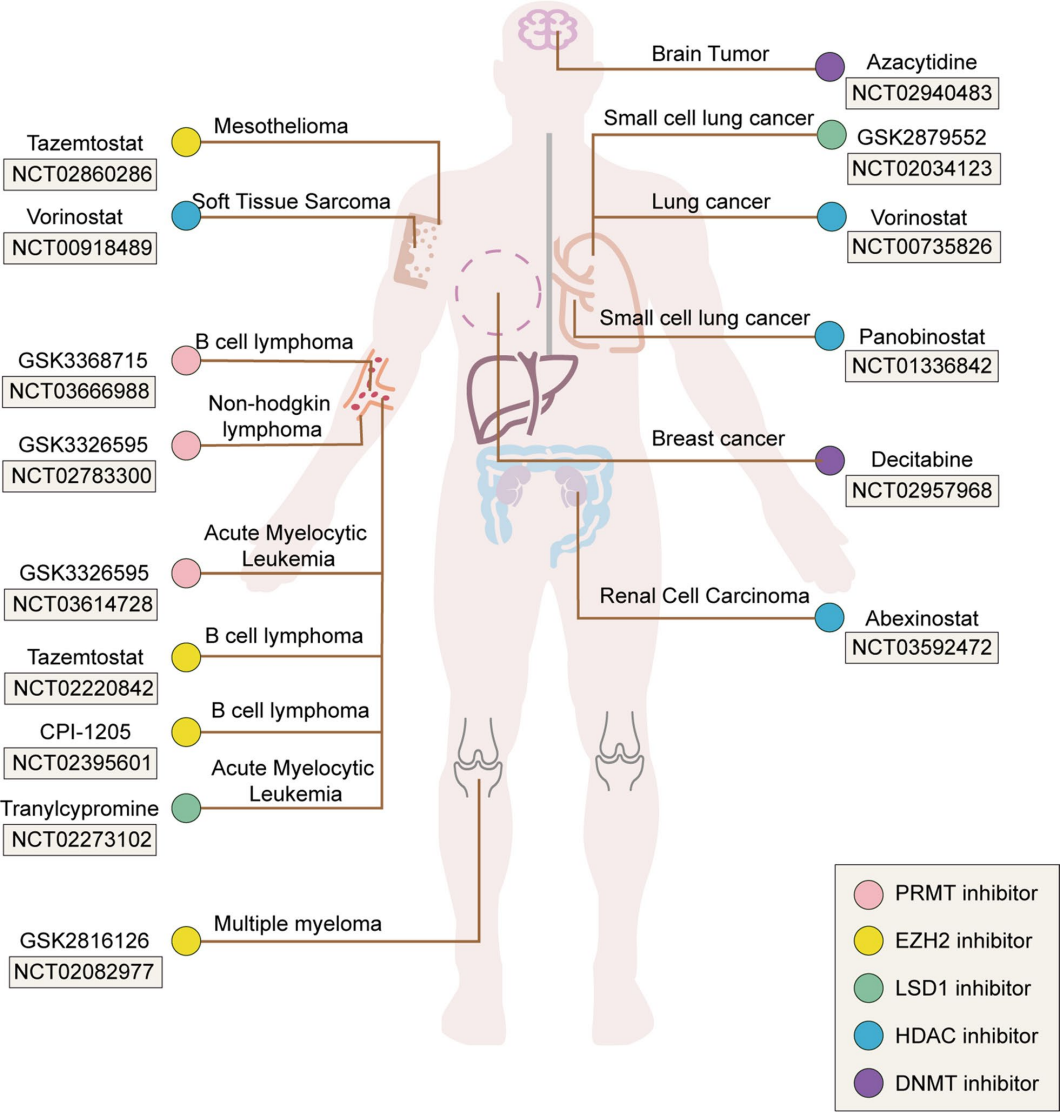
Targeting of certain cancers with 12 epi-drugs, including examples of clinical trial. The graph depicts the clinical trials of these PRMT, EZH2, LSD1, HDAC and DNMT inhibitors based on searches of the ClinicalTrials.gov database (https://clinicaltrials.gov/). Epi-drugs play an active role in hematological and solid tumors. However, epi-drugs that are mainly EZH2 inhibitors and PRMT inhibitors are more widely used in hematological tumors than in solid tumors. Moreover, the efficacy of EZH2 inhibitors and PRMT inhibitors in solid tumors is still limited. The effects of LSD1 inhibitors, HDAC inhibitors, and DNMT inhibitors on hematological tumors have also been analyzed in several clinical trials. However, clinical studies on their role in solid tumors such as lung cancer, breast cancer, and renal cancer have currently been successively carried out

Epi-drugs achieve anticancer effects mainly by targeting and inhibiting epigenetic modifying enzymes and adjusting abnormal epigenetic changes in tumors. Thus, epi-drugs participate in the interruption of the cell cycle and the activation of tumor suppressor pathways, which in turn induce programmed cell death in tumorigenesis. However, as targeted modulators, epi-drugs do not only target directly inhibiting enzymes; rather, it should also be determined whether the sensitivity to epi-drugs in different patients is based on biomarkers. Epi-drugs cannot exert their potential antitumor activity at the applicable dose in insensitive to the patients. In addition, the greatest obstacle faced during the development of epi-drugs or other types of drugs is the occurrence of adverse side effects. In the development of epi-drugs, it is necessary not only to find potential biomarkers, but also to ensure that epi-drugs exert their maximum efficacy at a minimum dose and to avoid the generation of toxic side effects. Among the above 12 epi-drugs, six epi-drugs have been approved by the FDA for clinical treatment, including mainly EZH2 inhibitors, HDAC inhibitors, and DNMT inhibitors. We display the overview of clinical trials of 12 epi-drugs in certain cancer types to reference the application on epi-drugs in clinical use, including aspects as target cancer type selection, or in-depth study of the anti-tumor mechanism (Fig. 5). Nevertheless, no PRMT inhibitors and LSD1 inhibitors have currently achieved an FDA approval. Further, there are still some contradictions regarding LSD1 inhibitors. Thus, there is controversy regarding the function of LSD1 as an oncogene or a tumor suppressor. The function of LSD1 inhibitors as anticancer epi-drugs has been recognized, but it needs to be further studied whether their targets exert anticancer effects by inhibiting LSD1.

In addition to the epi-drugs mentioned above that directly target on the epigenetic proteins, there is also a class of drugs that deserve our attention. These kinds of drugs are not typically regarded as "epigenetic" drugs. Although these drugs do not directly act on the epigenetic regulators, they still established epigenetic function. For example, a standard chemotherapy in glioblastoma treatment, temozolomide, is discovered to have strong influence on enhance of DNA methylation resulting in global gene silencing [122, 123]. For the long-term point of view, the CRISPR/Cas approach gives a different perspective to target silence the locus-specific epigenetic genes, which offer puissant possibilities correct epigenetic mutations in tumorigenesis and drug resistance [124]. The epigenetic editing technique can be used to fuse the catalytic domain of epigenetic enzyme and modulate the dysfunction of epigenetic signature at precise positioning of tissues [125]. Based on the CRISPR/Cas approach, epigenetic editing might present new insight into the use of epi-drugs and diminish side effects to a greater extent.

Although cancer monotherapy can minimize the toxic and side effects of epi-drugs, combination trials targeting multiple targets can have a therapeutic effect in malignant tumors due to the tumor heterogeneity. For example, panobinostat is approved by the FDA as a third-line drug for the treatment of MM, and its combination can produce a synergistic effect in the treatment of cancer. Thousands of clinical trials of epi-drugs are currently being carried out, many of which focus on the combination of epi-drugs with immunotherapy or traditional chemotherapeutic drugs. Combination therapy has undergone incremental development, and combination therapy may provide a basic reference for clinical medical administration. Although clinical trials in the direction of combination therapy are currently progressing well, there are only a few clinical trials for available predictive biomarkers of epi-drugs. For less studied biomarkers, clinical trials are relatively conservative. Researchers should not only study biomarkers in depth but also apply the studied biomarkers in the clinical practice and truly achieve precise drug treatment in the future.

## Data Availability

All data are included in the paper.

## Abbreviations

Epi-drugs: Epigenetic drugs
DNMTs: DNA methyltransferases
HMTs: Histone methyltransferases
HDMs: Histone demethylases
HDACs: Histone deacetylases
HDACs: Histone deacetylases
PRMT: Protein arginine methyltransferases
EZH2: Enhancer of zeste homolog 2
MTAP: Methionine phosphorylase
DLBCL: Diffuse large B cell lymphoma
FTIH: First time in humans
NHL: Non-Hodgkin’s lymphoma
MDM4: Murine double minute 4
MDS: Myelodysplastic syndrome
AML: Acute myeloid leukemia
SCLC: Small cell lung carcinoma
NSCLC: Non-small cell lung carcinoma
SAM: S-Adenosyl methionine
FDA: United States Food and Drug Administration
ARS: Artemisinin succinate
ALAS1: 5-Aminolevulinic acid synthase
HR: Homologous recombination
MM: Multiple myeloma
ECG: Electrocardiogram
UCK1: Uridine-cytidine kinase 1
ISR: Integrated stress response
OCT2: Organic cation/carnitine transporter 2
STS: Soft tissue sarcoma
